# COVID-19/SARS-CoV-2 Infection: Lysosomes and Lysosomotropism Implicate New Treatment Strategies and Personal Risks

**DOI:** 10.3390/ijms21144953

**Published:** 2020-07-13

**Authors:** Markus Blaess, Lars Kaiser, Martin Sauer, René Csuk, Hans-Peter Deigner

**Affiliations:** 1 Institute of Precision Medicine, Medical and Life Sciences Faculty, Furtwangen University, Jakob-Kienzle-Str. 17, D-78054 Villingen-Schwenningen, Germany; markus.blaess@web.de (M.B.); La.Kaiser@hs-furtwangen.de (L.K.); 2 Institute of Pharmaceutical Sciences, University of Freiburg, Albertstraße 25, D-79104 Freiburg, Germany; 3 Department of Anesthesiology and Intensive Care Medicine, University of Rostock, Schillingallee 35, D-18057 Rostock, Germany; martin.sauer@uni-rostock.de; 4 Department of Intensive Care Medicine, Hospital of Magdeburg, Birkenallee 34, D-39130 Magdeburg, Germany; 5 Fraunhofer Institute IZI, Leipzig, EXIM Department, Schillingallee 68, D-18057 Rostock, Germany; 6 Organic Chemistry, Martin-Luther-University Halle-Wittenberg, Kurt-Mothes-Str. 2, D-06120 Halle (Saale), Germany; rene.csuk@chemie.uni-halle.de; 7 Associated Member, Faculty of Science, Tuebingen University, Auf der Morgenstelle 8, D-72076 Tübingen, Germany

**Keywords:** SARS-CoV-2, COVID-19, lysosomotropic compounds, lysosome, cytokine storm, cytokine release syndrome, viral host cell entry, approved active compounds, lysosomotropism, cathepsin L

## Abstract

In line with SARS and MERS, the SARS-CoV-2/COVID-19 pandemic is one of the largest challenges in medicine and health care worldwide. SARS-CoV-2 infection/COVID-19 provides numerous therapeutic targets, each of them promising, but not leading to the success of therapy to date. Neither an antiviral nor an immunomodulatory therapy in patients with SARS-CoV-2 infection/COVID-19 or pre-exposure prophylaxis against SARS-CoV-2 has proved to be effective. In this review, we try to close the gap and point out the likely relationships among lysosomotropism, increasing lysosomal pH, SARS-CoV-2 infection, and disease process, and we deduce an approach for the treatment and prophylaxis of COVID-19, and cytokine release syndrome (CRS)/cytokine storm triggered by bacteria or viruses. Lysosomotropic compounds affect prominent inflammatory messengers (e.g., IL-1B, CCL4, CCL20, and IL-6), cathepsin-L-dependent viral entry of host cells, and products of lysosomal enzymes that promote endothelial stress response in systemic inflammation. As supported by recent clinical data, patients who have already taken lysosomotropic drugs for other pre-existing conditions likely benefit from this treatment in the COVID-19 pandemic. The early administration of a combination of antivirals such as remdesivir and lysosomotropic drugs, such as the antibiotics teicoplanin or dalbavancin, seems to be able to prevent SARS-CoV-2 infection and transition to COVID-19.

## 1. Introduction

Severe acute respiratory syndrome coronavirus 2 (SARS-CoV-2) has been identified as the disease-causing pathogen of the pandemic Coronavirus disease 2019 (COVID-19) [1]. Along with the outbreaks of severe acute respiratory syndrome coronavirus (SARS-CoV) causing the severe acute respiratory syndrome (SARS, 2002–2004), and Middle East respiratory syndrome coronavirus (MERS-CoV) causing the Middle East respiratory syndrome (MERS, 2012-current) [2], SARS-CoV-2 infection/COVID-19 is posing serious challenges to health care systems in the EU, the US, and many Asian countries.

SARS, MERS and COVID-19 are respiratory syndromes transmitted from person-to-person via close contact, singing [3], and probably airborne transmission (coughing) resulting in high morbidity and mortality in infected individuals. All three diseases are initially present as mild, influenza-like illnesses with fever, myalgia or fatigue, dyspnea, and cough. Progression to more severe symptoms is characterized by an atypical interstitial pneumonia and diffuse alveolar damage, ending in the acute respiratory distress syndrome (ARDS), the most severe form of acute lung injury. Alveolar inflammation, pneumonia, and hypoxic lung conditions, most likely accompanied by occurrence of syncytia (as seen in SARS patients [4]), lead to respiratory failure in multiple organ disease, and death in 50% of ARDS patients [2,5,6,7,8,9,10]. In China, the overall case-fatality rate (CFR) of SARS-CoV-2 infection/COVID-19 was 2.3% [8].

Human coronaviruses (HCoVs) including HCoV-OC43, HCoV-229E, HCoV-NL63, and HCoV-HKU1, as well as the highly pathogenic MERS-CoV (NC_019843.3, 30,119 bp RNA linear), SARS (NC_004718.3, 29,751 bp ss-RNA) and the newly emerging SARS-CoV-2 (isolate Wuhan-Hu-1, NC_045512.2, 29,903 bp ss-RNA) are currently classified as one of two genera in the family Coronaviridae [11,12,13]. Their most salient characteristics in common are: gene expression through the transcription of a set of multiple 30-nested subgenomic RNAs, expression of the replicase polyprotein via ribosomal frameshifting, unique enzymatic activities among the replicase protein products, a virion membrane envelope, and a multispanning integral membrane protein in the virion [12]. Typically, coronavirus infections are initiated by the binding of virions to specific cellular receptors such as ACE2 (SARS-CoV(-2)) [11,14,15,16] or DPP4 (MERS-CoV) [11] on the surface of host cells, culminating in the deposition of the nucleocapsid into the cytoplasm of the host cell where the viral genome becomes available for translation [12].

Research to identify active compounds for the treatment of SARS-CoV-2 viral infection/COVID-19 has focused, to date, on the virustatic agents ritonavir [17,18,19,20] (off-label use) and remdesivir [21,22,23,24,25,26] (GS-5734, compassionate use) or the antimalarial active compounds chloroquine [22,27,28,29] and hydroxychloroquine (plus azithromycin) [29,30,31,32,33] (off-label use), both of which are well-known immune modulators. Nevertheless, to date, the available clinical data are insufficient to recommend either for or against any antiviral or immunomodulatory therapy in patients with SARS-CoV-2 infection/COVID-19 or pre-exposure prophylaxis (PrEP) against severe acute SARS-CoV-2 [9].

As in SARS and MERS outbreak, the quest for suitable treatment options in COVID-19 initially has been focused on therapeutics with antiviral activities in HIV (lopinavir/ritonavir, darunavir/cobicistat, darunavir/ritonavir, and atazanavir), Ebola (remdesivir), Influenza A (umifenovir, favipiravir), and the disease-modifying antirheumatic drugs (DMARDs) chloroquine and hydroxychloroquine [9,34]. The efficacy data of active compounds provided by cellular, rodent, or nonhuman primate models of both highly pathogenic coronavirus infections SARS(-CoV) and MERS(-CoV) in earlier years have been neglected.

SARS-CoV, and very likely SARS-CoV-2 as well, is inducing cell death of host cells. Using the overexpression of individual SARS-CoV open reading frames (ORFs) to evaluate their intrinsic cytotoxicity, the following proteins have been reported to cause apoptosis in infected host cells: the 3CL-like protease; spike; ORFs 3a, 3b, and 7a; and the envelope (E), membrane (M), and nucleocapsid (N) proteins [35].

Apoptosis in mammalian cells is characterized by an increase in C_16_-ceramide [36,37]. Both can be blocked via lysosomotropic compounds such as NB 06, chlorpromazine, and imipramine [36]; apoptosis via chloroquine [38,39] and with its lysosomotropic characteristics C_18_-ceramide most likely as well. The lysosomotropic compound NB 06 down-regulates the expression of pro-inflammatory cytokines (e.g., IL-1B, IL-6 and IL-23A in LPS-stimulated macrophages [36] and desipramine protects against sepsis-induced cardiac dysfunction in a murine sepsis model [40].

Lysosomotropism is a commonly occurring and often neglected biological characteristic of small molecules leading to accumulation in lysosomes, which is present in addition to their intrinsic receptor-mediated or enzymatic pharmacological effects. Regardless of the medical indications for which they have been used, many (active) compounds possess lysosomotropic characteristics [36,41,42,43,44,45,46]. Therefore, they are potential active compounds for the treatment of SARS-CoV-2 viral infection of airway epithelial cells (type II pneumocytes), such as chloroquine in Sindbis virus infection [44]. Lysosomotropism is not unique to a particular type of cell; it affects all bronchoalveolar cells (including macrophages, dendritic cells, and granulocytes).

There is still a lack of drugs exhibiting pan-coronavirus antiviral activity, tackling host cell infection or the cytokine release syndrome (CRS)/cytokine storm syndrome in COVID-19. This increases the vulnerability of public health systems to a highly pathogenic coronavirus pandemic. Owing to the rapidly increasing number of cases, there is an urgent need for action in the field of therapy and prevention of SARS-CoV-2 infection/COVID-19. The therapies investigated and recommended to date represent a first step toward solving this immense challenge. Here, we outline possible prevention and treatment options based on lysosmotropic compounds, in combination with antivirals and recent findings on the SARS-CoV-2 disease. This concept would be easy to apply, even for patients at risk.

## 2. SARS-CoV-2, Host Cell Entry and Replication

### 2.1. SARS-CoV-2 and Cellular Receptor Angiotensin-Converting Enzyme 2 (ACE2)

SARS-CoV-2 is an enveloped non-segmented positive sense RNA human SARS-related coronavirus (SARS-CoV) [14] with a single-stranded RNA encoding at least four major structural proteins: spike protein (S), membrane protein (M), envelope protein (E), and nucleocapsid protein (N) [15]. The S glycoprotein comprises two functional subunits, which are responsible for fusion of the viral and host cell membranes (S_2_ subunit), and binding to the host cell receptor (S_1_ subunit), thus mediating entry into host cells [1]. S forms homotrimers protruding from the surface of the SARS-CoV-2 virus [1,47]. The receptor-binding motif (RBM; amino acids 437 to 508) is located in the receptor-binding domain (RBD) in the S_1_ subunit and has a high affinity toward human Angiotensin-converting enzyme 2 (ACE2; BRENDA:EC 3.4.17.23), a carboxypeptidase. The three-dimensional structure of the ACE2 receptor binding motif is similar in both SARS-CoV and SARS-CoV-2 [15]. ACE2 functions as a cellular receptor for SARS-CoV-2, allowing the virus to gain entry into ACE2 expressing host cells. It is widely distributed in cells (such as those of the lung (airwave epithelial cells), heart, liver, testis, kidney, brain, intestine (pancreas and colon), and several other tissues), and it circulates in blood vessels (circulating plasma ACE2) [1,15,16,48,49]. Nevertheless, SARS-CoV-2 mainly infects airway epithelial cells (pneumocytes) and macrophages; extrapulmonary spread of SARS-CoV-2 in ACE2 expressing tissues has also been observed [50].

### 2.2. SARS-CoV (-2) Mechanisms of Host Cell Entry

**Figure 1:**
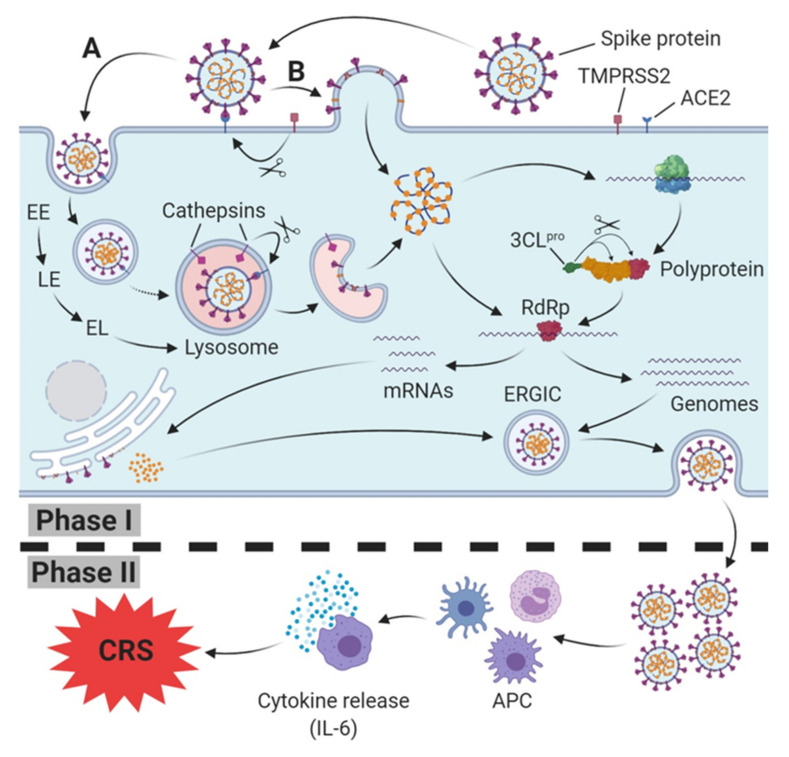
SARS-CoV(-2) host cell infection, replication (**Phase I)** and induction of immune response (**Phase II**). Spike protein (S protein) of SARS-CoV-2 binds to host cell membrane presented ACE2 (cellular receptor); it either enters the endocytic pathway (**A**) or fuses via TMPRSS2-mediated cleavage of the ACE2-S protein complex directly with the cell membrane (**B**). Traveling down the endosomal pathway, the maturation of early endosomes (EE), via late endosomes (LE) to early lysosomes (EL), and finally lysosomes, is accompanied by vacuolar acidification (indicated as red area). In late endosomes/lysosomes, the ACE2-S protein complex is cleaved via cathepsin L, resulting in the fusion of the viral and host cell membrane. After release into the cytoplasm, translation of viral RNA into the polyprotein takes place. Afterwards, the polyprotein is cleaved into several active non-structural proteins by the chymotrypsin-like protease subunit (3CLpro), including RNA depending RNA polymerase (RdRp). RdRp subsequently synthesizes progeny genomes and subgenomic mRNAs translated to structural proteins at the endoplasmatic reticulum. Both structural proteins and progeny genomes meet in the endoplasmic reticulum-Golgi intermediate compartment (ERGIC), resulting in the assembled SARS-CoV-2 virus. Finally, the nucleocapsid is released from host cell via exocytosis. In Phase II, SARS-CoV-2 is recognized and internalized by antigen presenting cells (APC), triggering an innate immune response which is accompanied by a release of cytokines, including Interleukin-6 (IL-6). In severe cases, a massive release of IL-6 leads to the cytokine release syndrome (CRS).

Both types of SARS-CoV engage their receptor, ACE2, on the host cell surface for host cell entry [4,15]. In cells that do not express trypsin-like proteases (human airway trypsin-like protease (HAT)) [51] on their surfaces, SARS-CoV enters the cytoplasm through endocytosis/endosomes and travels along the endocytic pathway. In lysosomes, the last compartment of the endocytic pathway, active cathepsin L (BRENDA:EC3.4.22.15; optimum pH 5.0–5.5 [52]), induces the fusion of SARS particles bound to ACE2 with host cells [53] by cleavage at the S1/S2 cleavage site of the SARS S (fusion) protein [54] (Figure 1).

The endosomal entry route of SARS particles bound to ACE2 and the subsequent maturation of early endosomes (EE) via late endosomes (LE) to endolysosomes (ELs), and finally lysosomes, has been substantiated by evidence that E64d (cysteine protease inhibitor) and bafilomycin A1 (inhibitor of vacuolar H^+^ ATPase (V-ATPase) (BRENDA:EC 3.6.3.6) and vacuolar acidification) block SARS-CoV(-2) S-protein-mediated endosomal entry [4,53,55]. The lysosomotropic cathepsin L inhibitor teicoplanin [45], a glycopeptide antibiotic, and Bafilomycin A1, via preventing (endo)lysosomal acidification inhibit cathepsin-L-induced fusion of SARS-CoV particles bound to ACE2 with host cells. The required lysosomal pH for fusion-activating S protein cleavage by cathepsin L can, therefore, be provided only by intact lysosomes. EE, LE, less acidic EL, lysosomes containing lysosomotropic compounds and cells with depleted ATP levels are unable to provide active cathepsin L.

In experimental in vitro conditions, trypsin can override the need for cathepsin-L-mediated cleavage, thereby shifting the virus to an endosome independent route of entry, possibly through the plasma membrane [56]. TMPRSS2 or TMPRSS11d, members of the transmembrane protease/serine subfamily (TMPRSS), have recently been reported to induce SARS-CoV(-2) fusion (S-protein priming) in cells [50,54,56].

### 2.3. SARS-CoV(-2) Mechanisms of Host Cell Entry by ACE2 Dependend Formation of Large, Multinucleate Syncytia

A hitherto neglected mechanism of cell–cell infection is the direct infection of a host cell by an infected neighboring host cell without the release of a complete virus from the infected host cell via exocytosis. During infection by some coronaviruses, including SARS-CoV(-2) [4], a fraction of S protein that has not been assembled into virions, ultimately reach the plasma membrane of infected host cells. Once it has reached the cell surface, the fraction of the S protein can cause the fusion of an infected host cell with adjacent, uninfected host cells, leading to the formation of large multinucleate syncytia [12]. This enables the spread of infection independent of the action of extracellular virus, thereby providing some measure to escape from immune surveillance [12]. Syncytia have been observed in Vero E6 cultures infected with SARS-CoV, in SARS-CoV-infected primates, and in SARS patients [4]. Both, SARS-CoV and SARS-CoV-2, are capable of cell–cell fusion without endocytosis to form a multinucleate cell, which results from multiple cell fusions of uninuclear cells. SARS-CoV(-2) S-Protein-related formation of multinucleate cells depends on the presentation of ACE2 on the host cell surface and is more pronounced in SARS-CoV-2 than in SARS-CoV [4].

### 2.4. Life Cycle of SARS-CoV-2

After the fusion of viral and host cells, SARS-CoV-2 and the deposition of the nucleocapsid into the cytoplasm the viral genome (ss-RNA) becomes available for translation into proteins [12,57]. The genetic material of SARS-CoV-2 consists of 11 open reading frames (ORF) [39], each encoding a variety of structural and non-structural proteins. Initially, the positive-sense genome (first mRNA of infection) is translated into the enormous replicase polyprotein, which then is autoproteolytically cleaved into several non-structural proteins (nsp1–nsp16) [39], and subsequently synthesizes progeny genomes and a set of subgenomic mRNAs via negative-strand intermediates. Subgenomic mRNAs are finally translated into structural proteins and accessory proteins [12]. According to recent findings, the replication of SARS-CoV-2 depends on the viral RNA-dependent RNA polymerase Enzyme (RdRp). Active SARS-CoV-2 RdRp consists of the nonstructural proteins nsp8 and nsp12 [58].

Indeed, this process is highly similar to previously identified coronaviruses (e.g., MERS-CoV, and SARS-CoV) and displays a potent therapeutic target suggesting the application of active compounds which have been proved to be effective in related coronavirus infections like MERS-Co-V. In contrast to other RNA viruses, however, SARS-CoV-2 has apparently no obstructive effect on host cell protein synthesis. SARS-CoV-2, however, seems to increase the hosts translation machinery, splicing, and nucleobase synthesis and seems to upregulate RNA modifiers, various nucleic acid metabolic pathways, and carbon metabolic pathways. Moreover, it is presumed that SARS-CoV-2 downregulates cholesterol metabolism [59]. The inhibition of translation (cycloheximide and emetine), spliceosome action (pladienolide B), glycolysis (2-Deoxyglucose), and nucleic acid metabolism (ribavirin), effectively inhibited viral replication in CaCo-2 cells. Each process is essential for SARS-CoV-2 replication and, therefore, offers potential therapeutic targets [59].

The host cell life cycle of SARS-CoV-2 is completed as proteins M, S, and E are inserted into the endoplasmic reticulum and transit to the endoplasmic reticulum-Golgi intermediate compartment (ERGIC). There, they coalesce with N protein capped, replicated progeny genomes (nucleocapsids). Now, the complete virus is assembled and exported to the plasma membrane in smooth-walled vesicles or Golgi sacs and released from the infected host cell via exocytosis [12,57].

## 3. Cytokine Release Syndrome (CRS)/Cytokine Storm Syndrome Related to SARS-CoV-2 Infection/COVID-19

SARS-CoV-2 mainly infects airway epithelial cells (pneumocytes) and macrophages [50]. In severe cases, SARS-CoV-2 is likely to cause both pulmonary and systemic inflammation, thus leading to multi-organ dysfunction (e.g., acute respiratory distress syndrome (ARDS), myocarditis, septic shock, sepsis, sepsis after bacterial superinfection, acute liver injury, and hepatitis) in high-risk populations [5,6,7,10,60]. Sepsis, a dysregulated host response (organ dysfunction) to an infection [61], was diagnostically confirmed in all of the 113 deceased individuals and was considered as the cause of mortality in all cases investigated. However, 66 (41%) of the 161 patients who recovered were diagnosed with sepsis. On the other hand, 46 (41%) among the 113 deceased individuals went into septic shock in the same study. Notably, there was no shock diagnosis in the group of recovered patients [6].

Concentrations of IL-2R, IL-8, TNFα, and IL-6 are significantly higher in deceased patients (72.0 (35.6–146.8) pg/mL than in recovered patients (13.0 (4.0–26.2) pg/mL), similar to the occurrence of sepsis, in which particular chemokines (including IL-6, IL-8, and IFNγ), CCL2, CCL3, and CXCL10, are involved in early stages [61]. Emerging evidence implicates IL6 as a central mediator of toxicity in cytokine release syndrome (CRS) [62]; therefore, these findings suggest that a rapid and severe deterioration during SARS-CoV-2 infection into COVID-19 is associated with CRS/cytokine storm syndrome [63,64].

## 4. Current Therapeutic Targets

**Figure 2:**
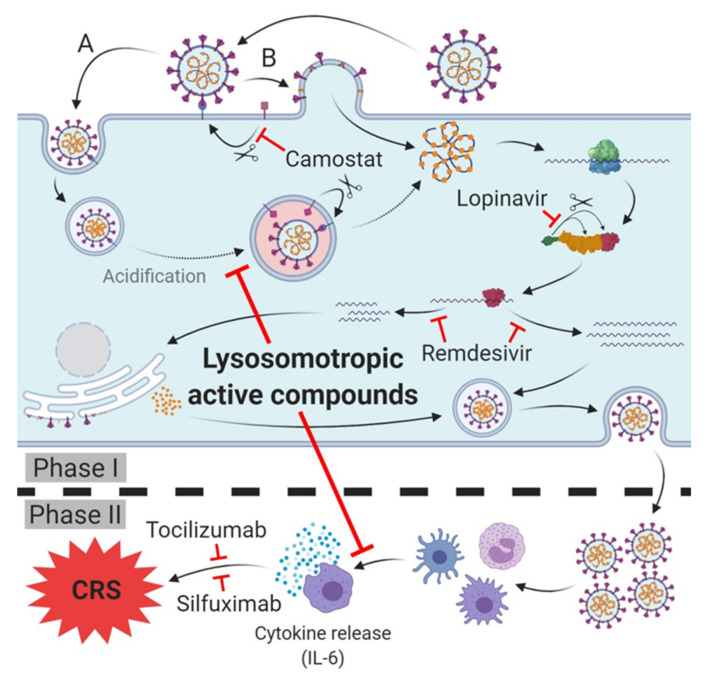
Therapeutic targets and lead compounds for treatment of SARS-CoV-2 infection/COVID-19. **Phase I** (host cell infection): TMPRSS2: Camostat inhibits protease activity, blocking SARS-CoV-2 host cell entry (variety (**B**)). 3CL^pro^: Lopinavir inhibits cleavage of the polyprotein, leading to diminished (activity of) subsequent non-structural proteins (nsp). RNA-dependent RNA polymerase (RdRp): Remdesivir inhibits synthesis of viral mRNA and progeny genomes. **Phase II** (cytokine release syndrome (CRS)/cytokine storm of COVID-19): Antibodies of IL-6 receptor (tocilizumab) or IL-6 (silfuximab) neutralize released IL-6 or block its receptor and prevent cytokine release syndrome (CRS)/cytokine storm and deterioration of SARS-CoV-2 infection into COVID-19. **Phase I** and **II**: Lysosomotropic active compounds prevent the acidification of endosomes, raise lysosomal pH and inhibit SARS-CoV-2 endosomal host cell entry (variety (**A**)). Expression of pro-inflammatory chemokines/cytokines in antigen presenting cells (APC) is diminished, thus preventing the cytokine release and CRS/cytokine storm and deterioration to COVID-19.

The most recent COVID-19 treatment guidelines (as of June 16, 2020) published by the NIH state that there is, at present, no drug or immunomodulatory therapy that has been proven to be safe and effective for treating SARS-CoV-2 infection/COVID-19. Various more or less promising strategies for the prophylaxis of SARS-CoV-2 infection and the treatment of SARS infection-CoV-2 and/or prevention of the transition to COVID-19 are conceivable. The current most promising ones are summarized below (Figure 2).

### 4.1. RNA-Dependent RNA Polymerase (RdRp)

The RNA-dependent RNA polymerase (RdRp, nsp12) is an essential nonstructural enzyme of SARS-CoV-2, highly conserved in various coronaviruses [12,24] and, therefore, a very promising therapeutic target against various coronavirus species. Nucleoside derivates are active compounds targeting RdRp, competing with endogenous nucleotides at the RdRp for incorporation into viral RNA, leading to an inhibition of viral replication through the premature termination of RNA transcription. Various compounds, including favipiravir, ribavirin, and remdesivir, are currently under investigation in SARS-CoV-2 infection [18,65,66]. However, remdesivir is currently rated as one of the most promising antiviral replication candidates, thus our review will address this particularly active compound [21,23,24,25,67,68].

Remdesivir (GS-5734) is an intravenous investigational nucleotide prodrug (monophosphoramidate) of an adenosine analog, metabolized to a 1’-cyano-substituted nucleoside analogue by an esterase-mediated separation of 2-ethylbutyl and the L-alanine residue [24]. Due to the 1′-CN residue, remdesivir and its metabolites exhibit high selectivity towards RdRp compared to human polymerases [58], and it bypasses the rate-limiting step of generation of nucleoside monophosphate.

In vitro and in vivo remdesivir demonstrated activity (based on animal studies) against SARS-CoV and MERS-CoV [20,26,68]. In prophylaxis or therapy, remdesivir also reduces MERS-CoV levels and lung injury in mice and MERS-CoV infection in rhesus macaques [68]. In addition, prophylactic application of remdesivir prevented MERS-CoV clinical disease in rhesus macaques [68]. A similar advantageous effect in SARS-CoV-2 is, therefore, very likely.

Initial reports on the in vitro activity of remdesivir against SARS-CoV-2 infection were published in February 2020 [22], simultaneously with reports on chloroquine. Since then, several clinical trials with remdesivir have been designed and registered within a short period of time; currently, about 22 clinical trials are recruiting patients for treatment with remdesivir in COVID-19 according to ClinicalTrials.gov, as of May 27, 2020.

Owing to limited oral bioavailability, remdesivir has to be applied intravenously. Remdesivir was evaluated in clinical trials on patients suffering from Ebola and COVID-19, starting with an initial dose of 200 mg on day 1, followed by an daily maintenance dose of 100 mg/d over a period of 5–10 days [23,66,69]. However, preliminary results on the clinical efficacy of remdesivir involving 237 patients in Hubei, China, were more than sobering, as remdesivir failed to demonstrate an advantage compared to placebo in time to clinical improvement, mortality, or time to clearance of virus in patients with severe COVID-19 [23]. Nevertheless, the authors state a limitation of interpretability due to small sample size (insufficient power) and recommend larger studies to detect the assumed differences in clinical outcomes. 

Most recently, preliminary results from a larger, multicentre clinical trial involving 1063 patients have been published [21]. These results demonstrate that remdesivir significantly lowers recovery time; however, mortality was not significantly reduced. Interestingly, further results indicate no difference if treatment is applied for a duration of 5 or 10 days [25].

In summary, it can be stated that the results from current clinical trials support the hypothesis of a measurable, but limited benefit of remdesivir in treatment of SARS-CoV-2 infections/COVID-19. Remdesivir turned out to be beneficial and is recommended for hospitalized patients with severe disease requiring supplemental oxygen, mechanical ventilation, or extracorporeal membrane oxygenation [9].

### 4.2. Chymotrypsin-Like Protease 3CL^pro^

The replication of SARS-CoV(-2) depends on the cleavage of polyproteins into an RNA-dependent RNA polymerase and a helicase [70] through 3-chymotrypsin-like protease (3CL^pro^, nsp5) and papain-like protease (PL^pro^, nsp3/4). 3CL^pro^ appears to be highly conserved in SARS-CoV-2 [71,72] and is the key protease responsible for the processing of early-formed polyproteins and displays an essential component in the life cycle of SARS-CoV-2 [12,73]. Several protease inhibitors designed for other virus families (e.g., HIV), exhibited in vitro activity against SARS-CoV and MERS-CoV, thus application for SARS-CoV-2 appears reasonable [74,75]. In this category of active compounds, lopinavir and ritonavir are currently listed as the most promising candidates [76].

Lopinavir and ritonavir are both inhibitors of HIV aspartate protease, essential for intracellular HIV assembly [34]. Usage of ritonavir resulted in a mutation of HIV protease, rendering ritonavir ineffective against HIV. In consequence, lopinavir (ABT-378) was developed as a way out, possessing a diminished interaction with the mutation site of HIV protease [77]. Both lopinavir and ritonavir, are currently approved as a combination therapy of HIV infections, where ritonavir inhibits CYP3A4 even in subtherapeutic dosage, resulting in higher plasma concentrations of lopinavir [34,75,76]. The inhibition of HIV protease results in the formation of immature virions and renders them noninfectious [76].

Lopinavir/ritonavir is an inhibitor of SARS-CoV 3CL^pro^ in vitro with a poor selectivity index resulting in higher than tolerable levels of the drug required in vivo to achieve meaningful inhibition [78]. Nevertheless, clinical trials with lopinavir/ritonavir in various settings were conducted very shortly after the beginning of the COVID-19 outbreak. The first enrolment of patients started at January 18, 2020, even though in vitro data on efficacy on SARS-CoV-2 was not available yet [17]. Initial clinical data failed to provide a clinical evidence of a benefit owing to lopinavir/ritonavir treatment, although reports published afterwards demonstrated an inhibitory impact on SARS-CoV-2 infected cells in vitro [17,19]. The clinical efficacy of lopinavir/ritonavir in SARS-CoV-2 infection/COVID-19 is rather limited and/or unverifiable, resulting in a recommendation against the use of lopinavir/ritonavir or other HIV protease inhibitors in COVID-19 [9].

If combined with interferon beta-1b and ribavirin, lopinavir/ritonavir demonstrated clinical efficacy by reducing recovery time from 12 (lopinavir/ritonavir) to seven days (interferon beta-1b/ribavirin/lopinavir/ritonavir) in early intervention (five days after onset) [18]. Interestingly, a combination of interferon beta-1b/ribavirin did not improve outcome in critically ill patients with MERS [79]. The beneficial effects observed in SARS-CoV-2 infection/COVID-19 are most likely to be attributed to triple therapy (of antivirals). Since in both studies no placebo groups were present for ethical reasons, a qualified conclusion on clinical efficacy compared to untreated patients is impossible.

### 4.3. Papain-Like Protease PL^pro^

Compared to 3CL^pro^ SARS-CoV PL^pro^ is able to strip ubiquitin and interferon-stimulated gene 15 (ISG15) from host-cell proteins to aid coronaviruses in their evasion of host-innate immune responses. Therefore, it is assumed that targeting PL^pro^ may have an advantage in inhibiting the dysregulation of signaling cascades in infected cells, leading to cell death in surrounding, uninfected cells [80]. Despite some promising model compounds [80], no approved active compound is available targeting PL^pro^ to date.

### 4.4. Interleukin-6 (IL-6)

Interleukin-6 (IL-6) is a pleiotropic, pro-inflammatory cytokine produced by a variety of cell types, including lymphocytes, monocytes, and fibroblasts. Infection by the related SARS-CoV induces a dose-dependent production of IL-6 from bronchial epithelial cells [81]. IL-6 plays a central role as a mediator of toxicity in the cytokine release syndrome CRS/cytokine storm, which is associated with severe cases of SARS-CoV-2 infection/COVID-19 [62,63]. COVID-19 is, in general, associated with heightened cytokine release, as indicated by elevated blood levels of IL-6 and C-reactive protein (CRP) [5,6,7].

Inhibition of IL-6 related signalling pathways via blocking IL-6 or IL-6 receptors (IL-6R) is a promising approach to prevent CRS/cytokine storm syndrome and rapid, severe, and serious deterioration during SARS-CoV-2 infection and transition to COVID-19 [6,82,83,84]. Current approved IL-6R antagonists include tocilizumab and sarilumab, approved for treatment of severe CRS in idiopathic arthritis, rheumatoid arthritis and giant cell arteritis [85,86]. Several clinical trials investigating the clinical efficacy of anakinra (IL-1R antagonist), siltuximab (anti IL-6 antibody that binds IL-6 prevents the binding of IL-6 to both soluble and membrane-bound IL-6R, inhibiting IL-6 signaling), tocilizumab and sarilumab (anti IL-6R antibody) in COVID-19 treatment are currently under way [87,88].

Initial reports of treatment with tocilizumab in severe cases of COVID-19 have yielded encouraging results to date. A small clinical trial with 20 COVID-19 patients proved the clinical efficacy of anti-IL-6R antibodies in severe cases of COVID-19. Tocilizumab effectively improves clinical symptoms (lowering body temperature to normal level within one day, significant lowering of C-reactive protein (CRP) after 5 days) and represses the deterioration of severe COVID-19 patients [89]. Sarilumab significantly decreases C-reactive protein (CRP) and mortality in critically ill, but not in severe, patients [9]. Results on siltuximab are, however, still pending.

### 4.5. Targeting the Metabolism of Host Cells

Modification of cellular metabolism by SARS-CoV-2 during replication is quite evident, as virally infected cells have higher anabolic processes and requirements to synthesize viral structural elements such as the viral lipid membrane [90]. While many DNA viruses appear to modify transcriptional regulation of key metabolic pathways, RNA viruses (including SARS-CoV-2) alter host cellular metabolism via post-transcriptional mechanisms [91,92,93]. At SARS-CoV-2, the latest research suggests a reduced cholesterol metabolism, an increased spliceosome, carbon, nucleic acid and glycerophospholipid metabolism, and an altered fatty acid (FA) profile in infected host cells [59,94]. Evaluation of patient sera identified lower levels of HDL-bound cholesterol, FA, tryptophan and choline, as well as higher levels of steroid hormones and bile acid derivates, while some alterations seem to be specific for severe COVID-19 [95,96]. Interestingly, supplementation of linoleic acid or arachidonic acid was able to inhibit HCoV-229E and MERS-CoV replication in vitro, suggesting endogenous metabolites as potential therapeutic agents for SARS-CoV-2 [94]. Furthermore, inhibition of glycolysis (2-Deoxyglucose), the spliceosome (pladienolide B), translation elongation (cycloheximide), as well as the deadlock of Scavenger receptor B type 1 (SR-B1) mediated lipid transfer between HDL and cells (BLT-1), led to inhibition of SARS-CoV-2 infection in vitro, further emphasizing the modification of cellular metabolism as a potential treatment approach for SARS-CoV-2 infection/COVID-19 [59,95].

Examples of current clinical trials on metabolism-modifying compounds or endogenous metabolites are statins (e.g., atorvastatin [97] and simvastatin (in combination with clopidogrel/rivaroxaban) [98]) for a reduction in cholesterol levels, as well as n3- fatty acids (e.g., eicosapentaenoic acid, gamma-linoleic acid) [99] to increase antioxidant species. Furthermore, a clinical trial (Phase 1) investigating 3,6-di-O-acetyl-2-deoxy-D-glucose (WP1122), a derivate of 2-deoxyglucose, has been announced by Moleculin Biotech recently; clinical data are unavailable to date.

Given the differences in the metabolic activities of various tissues [100], the inhibition of particular metabolic pathways should be carefully considered. The inhibition of glycolysis by 2-DG, for example, inhibits SARS-CoV-2 replication in CaCo-2 cells with an IC_50_ of 9.09 mM [59]. It should be noted that the modulation of myeloid differentiation of hematopoietic stem cells, as well as the inhibition of anti-inflammatory M2-macrophage polarization were observed already at 1 mM 2-DG [101,102]. Furthermore, concentrations of 0.6 mM 2-DG induced apoptosis in HUVECs and HMVEC-L [103]. In consequence, severe adverse events can be expected if 2-DG or its derivates are investigated in Phase 1 clinical trials or used in treatment of SARS-CoV-2 infection/COVID-19.

In sum, several reports suggest a promising potential in modifying single-metabolic pathways in SARS-CoV-2 infection/COVID-19; clinical data, however, are still missing. An extrapolation of in vitro studies on cell culture is very limited due to the distinct metabolic requirements of different tissues.

### 4.6. Targeting SARS-CoV-2 Host Cell Entry

Host cell entry of SARS-CoV-2 displays the initial step during host cell infection and viral replication; effective blocking at this early stage of disease is very likely to result in a successful prophylaxis and treatment. Several in vitro studies have been conducted to elucidate the entry route and therapy options for treating SARS-CoV infection [28,45,51,104]. Both SARS-CoV and SARS-CoV-2 bind via their spike protein on ACE2 on the host cell surface that functions as a cellular receptor to gain entry into host cells. After binding on ACE2, fusion of viral and host cell membrane is initiated by the protease-mediated cleavage of the ACE2-Spike protein complex, resulting in the activation of the S2-subunit and facilitating membrane fusion. The current knowledge suggests that this cleavage-mediated fusion can occur either immediately at the host cell surface by TMPRSS2 or, after passing the endosomal entry route within the lysosome catalyzed by lysosomal cathepsin L [25,50,56,105,106]. Therefore, the inhibition of both TMPRSS2 and cathepsin L display promising treatment approaches for SARS-CoV-2 infection/COVID-19. In this context, the approved TMPRSS2 inhibitors camostat mesylate [107,108] and nafamostat mesylate [109,110], as well as the lysosomotropic cathepsin L inhibitors chlorpromazine [111] and fluoxetin [112] are currently being investigated in upcoming clinical trials.

#### 4.6.1. TMPRSS2 Inhibitors

Nafamostat is a well-tolerated and approved synthetic protease inhibitor that inhibits various serine proteases such as Factor VIIa, Factor XIIa, kallikrein, thrombin, components of the complement system, and trypsin to treat (sepsis-related) disseminated intravascular coagulation (DIC) and acute pancreatitis (inhibition of trypsin) [113] As severe SARS-CoV-2 infection/COVID-19 is associated with coagulation abnormalities and comprises elements reminiscent of thrombotic microangiopathy and DIC [114], patients may benefit from nafamostat treatment in both the prophylaxis of SARS-CoV-2 infection and venous thromboembolism, probably related to coagulopathy. Owing to the poor bioavailability and very short elimination half-life of 8 min, nafamostat hast to be administered intravenously as a continuous infusion over a prolonged period (typically 90 min with 0.1 mg/kg/h) [109,110,113]. Although the blood level is easily controlled, nafamostat is therefore hardly suitable for SARS-CoV-2 infection prophylaxis but is useful for patients subjected to extracorporeal circulation [113] such as extracorporeal membrane oxygenation (ECMO) [115].

In contrast, camostat is an approved and orally bioavailable drug sharing the same inhibitor and target characteristics. Like nafamostat, camostat inhibits a variety of serine proteases, including trypsin, plasma kallikrein, thrombin and plasmin, and C1r- and C1 esterases [116,117]. The prodrug camostat is completely hydrolyzed during intestinal absorption to its active metabolite 4-(4-guanidinobenzoyloxy)-phenylacetic acid having an elimination half-life of 100 ± 40 min [118]. The very poor pulmonary recovery (about 1% in dog) of ^14^C-labelled camostat in a distribution experiment [118] poses the legitimate question of whether the pulmonary concentration in therapeutically dosage [109,110,116] is sufficient to generate the TMPRSS2 inhibition demonstrated in vitro [50,119] (i.e., providing prophylaxis of infection) in vivo as well, or whether the anticoagulant and antithrombotic effect of camostat can merely be utilized in SARS-CoV-2 infection/COVID-19. In vitro camostat mesylate has been observed to be 10-fold less active than nafamostat [119].

#### 4.6.2. Cathepsin L Inhibitors

Various selective cathepsin L inhibitors are known, however, none of these are clinically approved [120,121] and currently investigated. Nevertheless, some approved drugs seem to exhibit inhibitory effects for cathepsin L, including clofazimine, and rifampicin [120]. In cell culture experiments, the approved glycopeptide antibiotics (teicoplanin, telavancin, dalbavancin, and oritavancin) proved to be potent inhibitors of cathepsin L and were proved to prevent SARS-CoV infection in a dose-dependent manner within therapeutic dosage [53].

#### 4.6.3. Targeting SARS-CoV-2 Host Cell Entry/COVID 19 with Cathepsin L or/and TMPRSS2?

Interestingly, neither the inhibition of TMPRSS2 by camostat mesylate, nor the inhibition of cathepsin B/L by E-64d alone could prevent SARS-CoV-2 entry to TMPRSS2 transfected Vero cells, indicating that both key enzymes are mutually interchangeable in host cell entry [50]. These findings are supported by the fact that combined treatment with E-64d and camostat mesylate strongly prevented host cell entry; single treatment with lysosomotropic ammonium chloride elevating lysosomal pH, however, proved to effectively prevent SARS-CoV-2 host cell entry, independent of TMPRSS2 [50]. Unsurprisingly, the efficacy of nafamostat in blocking SARS-CoV-2 infection has been found to be cell-line-dependent and correlated to the expression of ACE2 and TMPRSS2 [119]. In ACE2 and TMPRSS2 expressing lung epithelium-derived cells (Calu-3 and H3255) nafamostat was extremely efficient (EC_50_ about 10 nM), whereas in non-TMPRSS2-expressing cells (293T and VeroE6) nafamostat was demonstrated to be hardly effective (EC_50_ about 30 µM) [50,119]. In this context, an EC_50_ within the range of 5–50 µM usually indicates an inhibitory effect grounded in lysosomotropism [36,44,53]; a hypothesis which is supported by the chemical structure and chemical characteristics of nafamostat.

Furthermore, the cell culture experiments revealed that TMPRSS2 inhibitors are solely efficient in SARS-CoV-2 infection if the viral host cell entry is exclusively mediated by TMPRSS2. As soon as the cathepsin-L-dependent endocytic pathway of infection (via ACE2 receptor) is participating, the effectiveness of TMPRSS2 inhibitors decreases rapidly [119].

Moreover, the efficacy of TMPRSS2 inhibitors is extremely dependent on the moment of application during the disease process. In TMPRSS2-expressing cell lines, EC_50_ was determined to be approximately 500-fold lower in pretreated cells than in non-pretreated cells (about 10 nM compared to 5 µM). If TMPRSS2 is not expressed, a dramatic loss in the efficacy of pretreatment occurs with an EC_50_ approximately 3000-fold higher (about 30 µM). Without pretreatment, efficacy is lacking (EC_50_ > 100 µM) [119].

A similarly pronounced dependence on certain enzymes, proteins, cell lines, and in particular on the time of application during the disease process, is assumed to be unlikely for lysosomotropic active compounds. The EC_50_ of active compounds is typically within 5–30 µM [36,41].

Consequently, raising lysosomal pH appears to display the more promising prophylaxis and treatment approach for SARS-CoV-2 infection/COVID-19.

## 5. Lysosomotropic Active Compounds

Lysosomotropic compounds are small molecules selectively gathering in lysosomes, regardless of their chemical nature or mechanism of uptake [43]. Typically, they are weak organic bases (pKa > 6, lipophilic) that easily penetrate, uncharged, the lysosomal membrane and are protonated and consequently trapped in the lysosome lumen [36,41]. Intralysosomal concentrations attain right up to a hundredfold of the cytosolic concentrations [122]. Lysosomotropism is therefore a biological characteristic of active compounds that is independent of their pharmacological effects.

Because lysosomotropic compounds accumulate in lysosomes, they increase the lysosomal pH from 4.5–5 to 6–6.5 [123]. Lysosomotropic effects (IC_50_) can be determined, and compounds can be screened by quantifying the displacement of Red DND-99 (LysoTracker) from lysosomes [124,125]. Effective compounds display a displacement IC_50_ of approximately 10 µM, which is in the range of the values for several receptor-mediated or enzyme inhibitory effects [36,44,126]. Most lysosomal enzymes are inactivated through an increase in the lysosomal pH beyond their optimum pH range (pH 4.5–5.5).

Lysosomotropic characteristics may diminish target specificity if the target is located in lysosomes; e.g., a nitrogen-containing lipophilic selective cathepsin K inhibitor in cell culture experiments results in an apparent increase in inhibitor potency against antitarget enzymes (cathepsin B, L, and S) present in lysosomes [46].

To date, lysosomotropism has been of scientific interest for its association with the occurrence of sometimes severe adverse effects during the application of particularly active compounds. Lysosomotropism, in combination with dysfunction in elongation of very long-chain fatty acids, is responsible for severe adverse effects when used orally or topically [127], in some cases such as hydroxychloroquine (rash or itching) [128], sertraline (exanthematous pustulosis) [129], and terbinafine (Lupus erythematodes or exanthematous pustulosis) [130]. Lysosomotropism appears at concentrations in the micromolar range; nevertheless, most drugs exhibit their desired primary pharmacological effects at low concentrations.

In fact, various well-known approved active compounds for various indications (psychotropic, antihypertensive, and antimycotic) share lysosomotropic characteristics. To date, amitriptyline, amlodipine, astemizole, benzatropine, bepridil, chlorpromazine, chlorprothixene clomiphene, desipramine, doxepine, fluoxetine, imipramine, maprotiline, norfluoxetine, nortriptyline, paroxetine, promazine, promethazine, sertraline, terfenadine, and triflupromazine have been classified as lysosomotropic compounds (Table 1) [41,42].

## 6. Lysosomotropic Active Compounds in SARS-CoV-2 Infection In Vitro

### 6.1. Lysosomotropic Active Compounds in SARS-CoV-2 Infection

The subsequent maturation and acidification of early endosomes (EE) via late endosomes (LE) to endolysosomes (ELs), and finally lysosomes, is required for fusion-activating S protein cleavage by cathepsin L. The required lysosomal pH can therefore be provided only by an intact lysosome. Lysosomes containing lysosomotropic compounds and cells with depleted ATP levels are unable to provide active cathepsin L, which plays a crucial role in SARS-CoV-2 infection of host cells and subsequent dissemination. In general, cathepsin L can be inactivated through selective but not clinically approved inhibitors [46,121], or alternatively by approved lysosomotropic compounds in off-label use.

Chloroquine is a lysosmotropic active compound [38] already investigated in the context of SARS-CoV. Interestingly, chloroquine exerts antiviral effects during pre- and post-infection conditions [28]. Furthermore, chloroquine impairs the terminal glycosylation of ACE2 (the cellular receptor of SARS-CoV) at anti-SARS-CoV concentrations and inhibits viral entry (fusion). The levels of host-cell-surface ACE2 have been found to remain unchanged. This activity may affect endosome-mediated viral fusion with host cells and subsequent viral replication or assembly, thus releasing the virus and abrogating the infective process [28]. In sum, these findings suggest that both prophylactic and therapeutic effects of lysosomotropic compounds are very likely.

### 6.2. Accumulation of Lysosmotropc Active Compounds in Airway Epithelial Cells and Lung Tissue

Because the respiratory tract is the gateway for SARS-CoV-2 infection, the accumulation of lysosmotropic active compounds in the upper airway and airway epithelial cells is vital for the protective effect, regardless of the route of application. An extensive accumulation of various amines with a pKa > 8.5 in perfused lung tissue was first reported in 1974 [133]. Of the compounds in Table 1, the extensive accumulation of imipramine and chlorpromazine in isolated perfused lung tissue and of imipramine in alveolar macrophages has been demonstrated [133,134,135]. Therefore, it is reasonable to assume that the additional active compounds in Table 1 will also accumulate in perfused lung tissue and provide a protective effect on SARS-CoV-2 infection/COVID-19.

### 6.3. Chloroquine and Hydroxychloroquine

As long ago as 1984, effects of the weak base and lysosomotropic compound chloroquine in Sindbis virus infection in BHK-21 cells were investigated. In established infections, chloroquine was found to inhibit the synthesis of viral RNA when added early in the process of pathogenesis [44]. Years later, in 2005, an in vitro study, focusing on the effects of chloroquine during pre- and post-infection periods, has provided profound insight into the progress of SARS-CoV infection. Chloroquine has been found to abolish SARS-CoV infection in Vero E6 cells, starting from 0.1 μM chloroquine in a dose-dependent manner, whereas concentrations of 10 µM chloroquine completely inhibit SARS-CoV infection. As little as 0.1–1 μM chloroquine decreases the number of virus antigen-positive host cells by 50%. The IC_50_ of SARS-CoV is 4.4 ± 1.0 μM [28]/8,8 µM [104], and that of SARS-CoV-2 is 6.9 µM [32].

After the outbreak of SARS-CoV-2/COVID-19 in late 2019, successful in vitro experiments from 2005 that tested the effects of chloroquine on SARS-CoV infection of Vero E6 host cells [28] were repeated, now targeting SARS-CoV-2. In addition to chloroquine, the less toxic active compound hydroxychloroquine was tested in the same experimental setting [29]. In SARS-CoV-2 infection, as in SARS-CoV infection, chloroquine displays similar inhibitory activity. Hydroxychloroquine shows a somewhat (approximately two times) lower inhibitory effect on viral infection of Vero E6 cells than chloroquine (determined as EC_50_ per multiplicity of infection (MOI)), and thus may be a viable treatment option.

### 6.4. Glycopeptid Antibiotics

Teicoplanin and dalbavancin are solely intravenously applicable, clinically approved glycopeptide antibiotics. Both compounds display a pronounced inhibitory effect against SARS-CoV infection (fusion) in HEK293T cells (IC_50_ teicoplanin: 3.76 ± 1.1 µM; dalbavancin; 9.64 ± 1.3 µM) but not against free cathepsin L enzyme (IC_50_ > 200 µM) [45]. This discrepancy indicates lysosomotropic effects of teicoplanin and dalbavancin. Similarly to lysosomotropic aSMase inhibitors [36], lysosomal enzymes are generally inhibited by the tested compounds only in intact cells [45].

Similarly to other lysosomotropic compounds, both active compounds are accompanied by pruritus, urticaria, and rash as undesired adverse effects, thus indicating lysosomotropic characteristics. Furthermore, oritavancin (IC_50_ 4.96 ± 1.2 µM) and telavancin (IC_50_ 3.45 ± 1.2) are very promising approved glycopeptide antibiotics with similar characteristics [45]. A unique feature of glycopeptide antibiotics is that, if they are used off-label as lysosomotropic compounds, they retain their initial adverse effect profiles. In off-label use, the benefit–risk profile is indistinguishable from that in authorized applications

### 6.5. Lysosomotropic Approved Small Molecules

The group of lysosomotropic small molecules comprises approved active compounds (pharmaceuticals) from a wide range of indications that have been tested for their lysosomotropic characteristics and/or their protective effect on SARS-CoV(-2) infected cells (Vero E6) [28,29,36,41,42,45,131,132]. The majority of the active compounds listed in Table 1 have an influence on neurotransmitters ((reuptake) inhibitors). In addition, there are tyrosine kinase inhibitors, ovulation inducers, estrogen receptor antagonists, antibiotics, antimycotics, and other indications within this group. All compounds of Table 1 share in one or more aliphatic protonatable, endocyclic, or exocyclic nitrogen in common, responsible for their lysosomotropism.

However, in the case of systemic administration of the active compound, the main indications must be considered because they may represent undesired adverse effects in an anti-viral off-label use, and therefore should not be ignored. According to current knowledge, the inhibition of cathepsin-L-dependent viral entry (fusion) into host cells can be obtained only through off-label use of the active compounds listed in Table 1.

### 6.6. Lysosomotropic Small Molecules for Research Purposes

Besides the approved lysosmotropic active compounds, there are other lysosmotropic small molecules such as NB 06 and NB 19 available as tools for studying the biological effects of lysosomotropism and lysosome-dependent signaling pathways [36].

## 7. Tackling the CRS/Cytokine Storm Syndrome in COVID-19

The hypothesis that lysosomotropic compounds such as chloroquine, owing to overactivation of the immune system triggered by SARS-CoV-2 infection, are able to suppress the CRS/cytokine storm syndrome and to attenuate the transition from mild to severe [32], is supported by the results of gene expression experiments with the small molecule model compound NB 06, in a setting addressing the effects of lysosomotropic compounds in LPS-induced inflammation in monocytic cells [36]. NB 06, like chloroquine, modulates the gene expression of the prominent inflammatory messengers IL-1B, IL-23A, CCL4, CCL20, and IL-6; likewise, it has beneficial effects in (systemic) infections involving bacterial endotoxins, such as LPS, by targeting the TLR4 receptor pathway in sepsis. These findings are consistent with the reported inhibitory effects of hydroxychloroquine on antigen processing and MHC class II presentation, interference with Toll-like receptor (TLR) signaling (TLR9 and TLR7), and inhibition of TNFα, IFNα, IL-6, and CCL4 production [136]. The host inflammatory response to an infection, via TLR4, can induce a cytokine storm, thus resulting in acute pulmonary inflammation. Targeting the cellular TLR4 signaling pathway and inflammatory cytokine production has been demonstrated to be successful in vitro as well as in vivo in TLR4-null mice [137]. In contrast, resatorvid (TAK-242), a small molecule TLR4 antagonist, does not suppress cytokine levels in patients with sepsis and shock or respiratory failure [138].

NB 06 and the clinically approved lysosomotropic compounds listed in Table 1 might, therefore, serve as valuable active compounds to prevent or mitigate the cytokine storm in the lungs after viral (SARS-CoV-2) infection of airway epithelial cells.

## 8. Modular Prophylaxis and Treatment in SARS-CoV-2 Infection/COVID-19

COVID-19 originates from a SARS-CoV-2 infection that could not be tackled successfully by the immune system. An effective one-step or single active compound therapy has not yet been identified. It is, however, reasonable to divide the development of COVID-19 into two phases: an initial viral infection phase 1 and a phase 2 encompassing the development of the cytokine storm/CRS and transition to COVID-19 and/or the bacterial secondary infection. Each of the two phases can be selectively addressed. The goal of a successful therapeutic strategy is to provide the immune system with support in fighting the initial viral infection. The viral load of the infected host cells and the number of cells in which viral replication occurs should be as low as possible.

### 8.1. Phase 0—Reducing the Viral Load (in Host Cells)

(Hydroxy)chloroquine [22,28,29] and lysosomotropic active compounds, in general, increase endolysosomal pH, inhibiting the fusion of the SARS CoV-2 particles bound to ACE2 and the host cell membranes required for release of the viral genome. Consequently, lysosomotropic active compounds are very well suited for prophylaxis through the inhibition of cathepsin-L-mediated cleavage. Alternatively, TMPRSS2 inhibitors may be applied if it is ascertained that TMPRSS2 is expressed and is participating in the process of infection and if the application is started early, immediately at the onset of the disease process, or for prophylaxis.

### 8.2. Phase 1—Viral Infection and Replication

The viral infection (phase I) can be treated successfully by antiviral active compounds like remdesivir within a limited period (5–6 days), shortly after the symptoms emerge and viral shedding occurs [139]. As soon as the infection initiates a CRS/cytokine storm, it is likely that the transition towards COVID-19 (phase II) or bacterial secondary infections occur.

### 8.3. Phase 1—Host cell–Host Cell Viral Infection by Viral S Protein without Viral Exocytosis Forming Multinucleate Syncytia

The SARS-CoV-2-related formation of multinucleate infected cells is more pronounced in SARS-CoV-2 than in SARS-CoV [4] and independent of the endolysosomal entry route. Cell–cell fusion may be triggered by a HAT-mediated fusion of the S protein ACE2 complex of the non-virus-infected cell with the virus-infected cell. This is where the approved TMPRSS2 inhibitors camostat and nafamostat might be useful. It is, however, questionable whether a sufficiently effective concentration of both active compounds can be achieved on the surface of the affected cells. Whether a benefit of camostat in combination with hydroxychloroquine can be obtained is the subject of a scheduled clinical trial [107].

C_2_-, C_6_- and C_18_-ceramides are effective in triggering exocytosis in rat PC12 cells [140], C_6_-ceramide triggers endocytic vesicles in murine 3T3-L1 cells [141], and inhibits glycoprotein traffic through the secretory pathway in vesicular stomatitis virus-infected CHO cells [142]. Of particular interest in SARS-CoV-2-related cell–cell fusion is the triggering effect of C_18_-ceramide on exocytosis of fractions of viral S protein. Analogously to C_16_-ceramide, lysosomal synthesis and an increase in C_18_-ceramide can be prevented by lysosomotropic active compounds [36,127].

### 8.4. Phase 2—Transition to COVID-19 and/or the Bacterial Secondary Infection

Lysosomotropic active compounds, however, are very likely to prevent a CRS/cytokine storm and the transition from phase 1 to 2. The incidence of CRS/cytokine storm associated with secondary bacterial infections is likely to be reduced by using antibacterials with lysosomotropic characteristics such as teicoplanin and dalbavancin in appropriate systemic drug levels.

### 8.5. Prophylaxis for High Risk Patients

As mentioned previously, in prophylactic application, remdesivir was proved to prevent MERS-CoV clinical disease in rhesus macaques [68], and probably in SARS-CoV-2 as well. It is evident that the early administration of a combination of remdesivir and teicoplanin, dalbavancin, oritavancin, or telavancin could be able to prevent a transition from SARS-CoV-2 to COVID-19.

## 9. Lysosomotropic Active Compounds in Clinical Trials

A large number of approved, tolerated, and well-known active compounds possess lysosomotropic characteristics, including (hydroxy-)chloroquine, chlorpromazine, and fluoxetine which are undergoing or will soon begin clinical trials for the treatment of SARS-CoV-2 infection/COVID-19.

### 9.1. Chloroquine and Hydroxychloroquine

Chloroquine (phosphate) and hydroxychloroquine (sulfate) are both DMARDs. In cell culture experiments, hydroxychloroquine has been demonstrated to prevent SARS-CoV-2 infection in vitro [22,29,32]. Recent clinical trials have demonstrated that oral hydroxychloroquine (600 mg per day) is significantly associated with a reduction in/disappearance of viral load in nasopharyngeal samples in patients with SARS-CoV-2 [31].

#### 9.1.1. Chloroquine

The drug profile of chloroquine is fraught with problems including severe unacceptable adverse effects such as dysrhythmias, often occurring in combination with other drugs such as azithromycin that prolong the QTc interval in and beyond the therapeutic margin [9]. Chloroquine and desethylchloroquine (active metabolite) concentrations decline very slowly with a terminal elimination half-life of 45 ± 15 days [38,143,144]. Modeling of chloroquine levels in lung (300 mg, ten days, twice per day) displayed a slow increase after the initial dose, and a delayed attainment of steady state on day 10 [33].

For a safe and reliable therapy and dosage of choroquine, the recommendations of the US Centers for Disease Control and Prevention (CDC) malaria treatment guidelines can be considered: 600 mg base orally at once, followed by 300 mg base orally at 6, 24, and 48 h [145,146]. Consequently, high-dose chloroquine (600 mg twice daily for 10 days) versus low-dose chloroquine (450 mg twice daily for 1 day followed by 450 mg for 4 days) leads to a significantly higher mortality and prolonged QTc interval in the high dosage arm of the study, finally leading to a termination of high dosage treatment [147]. Concurrently azithromycin (500 mg once daily for 5 days) reinforces the disappointing outcomes. Due to a missing placebo arm, no benefit in treatment of COVID-19 could be evaluated in this study.

Following the therapy recommendations of malaria prophylaxis [146], chloroquine, at present, is being investigated as a prophylaxis/protection of health workers (dosage regimen: low-dose (300 mg chloroquine base weekly), medium-dose (300 mg chloroquine base twice weekly), and high-dose (150 mg chloroquine base daily) [148] and versus hydroxychloroquine/placebo [149].

#### 9.1.2. Hydroxychloroquine

Hydroxychloroquine is better tolerated than chloroquine, sharing a common adverse effect profile. Terminal elimination half-life is 41 ± 11 days, within the range of chloroquine [38,136]. Dosage recommendations of CDC in malaria treatment guidelines (620 mg base orally at once, followed by 310 mg base orally at 6, 24, and 48 h) are considered as the upper limit of a save dosage regimen. In placebo-controlled treatment and/or prevention/prophylaxis clinical trials typically 400 mg (orally BID (day 1)), 200 mg (orally BID (days 2–5)) were applied [150,151,152], whereas in the treatment of (mild) COVID-19 600 mg daily for 7 days is the favorite [153]. As with chloroquine, in lung concentration modeling, the highest concentrations in lungs are obtained at day 10 with a variety of possible dosage regimens [33]. The results of the clinical trials are still pending. Preliminary results of a clinical trial investigating the effect of hydroxychloroquine on novel coronavirus pneumonia (COVID-19) [30,154] demonstrated some benefits of hydroxychloroquine in duration of fever and cough (one day less), progression of disease, moderate or significant improvement in chest CT scan.

#### 9.1.3. Disadvantages of Chloroquine and Hydroxychloroquine

Both chloroquine and hydroxychloroquine, share a similar toxicity profile, although hydroxychloroquine is better tolerated and has a lower incidence of toxicity than chloroquine; both provoke cardiac adverse effect (QTc prolongation, Torsade de Pointes, ventricular arrythmia, and cardiac deaths), and the development of rashes, pruritus and retinopathy [9,38,136]. In particular, the long elimination half-lives of both compounds with 30–60 days and varying substantially from one person to another which, impede therapy management and increase the risk of unacceptable serious adverse effects.

Oxidative stress (e.g., enhanced ROS levels and disturbance of antioxidant defense) has been demonstrated in experimental animal models of SARS [155]. Thus, intravascular hemolysis and methemoglobinemia has been observed in SARS-CoV-2 patients with Glucose-6-phosphate dehydrogenase (G6PD) deficiency [156], probably induced by oxidative stress in SARS-CoV-2 infection/COVID-19. Moreover, TMPRSS2 and cathepsin L, chloroquine and hydroxychloroquine are supposed to trigger severe drug-induced haemolytic anaemia in G6PD-deficient patients [157,158,159]. Although hydroxychloroquine is considered to be safe in therapeutic doses in class II or III G6PD deficiency, an increased risk of severe adverse effects is supposed to be present in patients suffering from SARS-CoV-2 infection/COVID-19. 

G6PD deficiency is one the most enzyme disorders worldwide, affecting 400 million people in Africa, Asia, the middle east, and the Mediterranean, and, owing to migration, however, now also in the Americas and Northern Europe [158]. Oxidative stress resulting from viral infection implies a high risk of serious adverse effects in G6PD-deficient patients, if treated with (hydroxy)chloroquine. Within this population the use of both hydroxychloroquine and chloroquine is rather contraindicated.

This, together with a lack of treatment efficiency, requires a quest for alternative lysosomotropic active compounds with a lower elimination half-life, better G6PD tolerance, and toxicity.

### 9.2. Chlorpromazine and Fluoxetine

Various active compounds have lysosomotropic characteristics (Table 1). Chlorpromazine displayed antiviral effects in vivo [131] and protective effects on COVID-19 in patients of a psychiatry hospital compared to health care workers in the same facility. Additional feedback from other psychiatry units in France, Italy and Spain, sustains the hypothesis that particular drugs may have beneficial effects in the prophylaxis of SARS-CoV-2 infection and transition to COVID-19. Consequently, chlorpromazine is a promising candidate in COVID-19/CRS treatment and a clinical trial has been enrolled [111]. Fluoxetine is another lysosomotropic compound being tested for its efficacy on SARS-CoV-2 infection/COVID-19 [112]. Chlorpromazine (11.5 h) [160] and fluoxetine (1 to 3 days after acute administration and 4 to 6 days after chronic administration) [161] have a significantly shorter elimination half-life than chloroquine/hydroxychloroquine and are less toxic.

In case of treatment of people without mental illness, however, a premature termination of this clinical trial due to the severe side effects of chlorpromazine is extremely likely. This raises the question of how to handle this issue to provide well-tolerated lysosomotropic drugs in SARS-CoV-2 infection.

### 9.3. Blend of lyLosomotropic Active Compounds

Several clinical trials are currently conducted to evaluate the clinical efficacy of lysosomotropic active compounds in COVID-19 [111,152,162]. Of particular interest is, focusing on the handling of possible severe adverse effects of hydroxychloroquine which investigates a mixture of two lysosomotropic compounds (hydroxychloroquine (200 mg twice a day instead 400 mg) and famotidine (360 mg/d intravenously)) in COVID-19 [162].

## 10. Hypothesis Regarding Lysosome Related (Skin) Diseases, Lysosomotropism, SARS-CoV-2 Carriers, Spreaders and Non-Infectable Humans

The lysosomes, and particularly cathepsin L, appear to play a key role in SARS-CoV-2 infection of host cells. To provide maximum cathepsin L activity and thus maximal cleavage capacity of viral S protein, properly functioning (endo)lysosomes are essential. If the lysosomal pH increases, cathepsin L activity diminishes, thereby decreasing the rate of cleavage, fusion, and infection.

Two scenarios can trigger an increase in lysosomal pH: a lysosomal proton pump breakdown or the administration/presence of lysosomotropic compounds. To date, a breakdown of the vacuolar H^+^ ATPase (V-ATPase) cannot be triggered by clinically approved active compounds. There is evidence, however, that in various skin disorders (e.g., psoriasis vulgaris, atopic dermatitis, exanthematous pustulosis, and pustular psoriasis), the function of the V-ATPase is more or less restricted by ATP deficiency caused by oxidative stress in cells (keratinocytes) [127]. Therefore, depending on the severity of the skin disease, individuals might not be infected (high ATP depletion and V-ATPase breakdown), whereas with moderate ATP depletion, individuals may have a much more moderate course of disease. Infection (fusion) of the airway epithelial cells is then assumed to be reduced or to be impossible.

**Figure 3:**
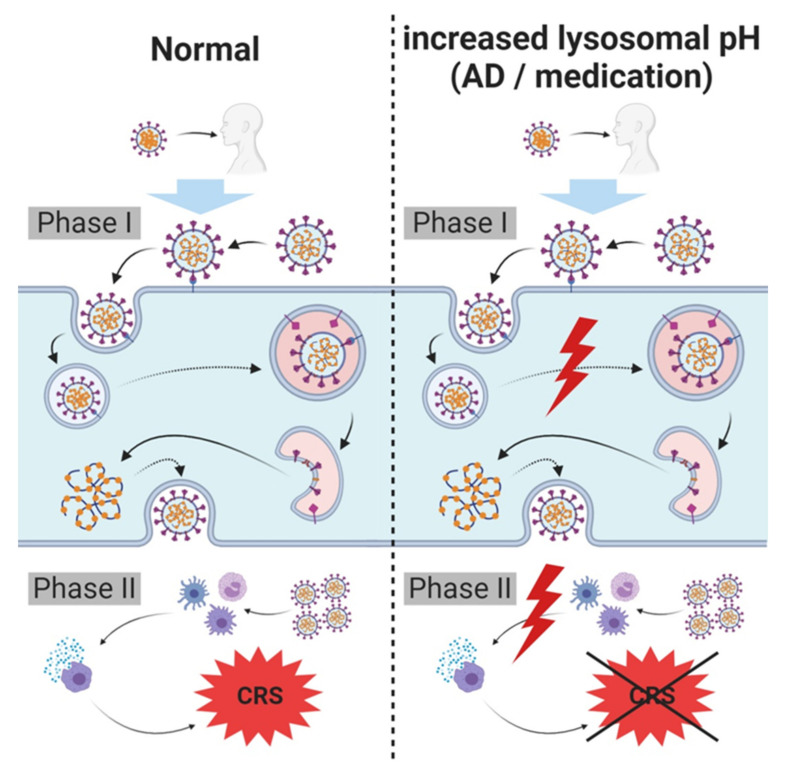
Hypothesis regarding lysosome related (skin) diseases, lysosomotropism, SARS-CoV-2 carriers, spreaders and non-infectable humans. SARS-CoV-2 infects host cells and triggers cytokine release requiring intact lysosomes. In case of an increased lysosomal pH, cellular entry and severe cytokine release (cytokine release syndrome (CRS)/cytokine storm) are inhibited, diminishing symptoms of infection and/or severity of COVID-19; complete prevention of infection is possible. An increased lysosomal pH in these individuals may either be the result of a breakdown of lysosomal proton pump (vacuolar H^+^ ATPase (V-ATPase)), resulting from various disorders (e.g., atopic dermatitis (AD) or psoriasis vulgaris), or due to the administration of lysosomotropic compounds for other pre-existing conditions.

Individuals receiving lysosomotropic active compounds in high dosages (e.g., sertraline (200 mg/d), terbinafine (250 mg/d), paroxetine (60 mg/d), or amitriptyline (150 mg/d) (given as maximum daily doses of each active compound)) exhibit a drug-induced increase in lysosomal pH. Cathepsin L is inactivated by lysosomotropic active compounds, and a significant decrease in viral load or no SARS-CoV-2 infection (fusion) of airway epithelial cells is expected (Figure 3).

Depending on the increase in lysosomal pH, individuals with these characteristics may have a lower or no risk of acquiring SARS-CoV-2 infection, as compared with the risk of untreated or healthy individuals with intact (endo)lysosomes. The severity of the infection (viral load) may also be reduced in these individuals. Observations of protective effects of chlorpromazine on COVID-19 in patients in a psychiatry hospital compared to health care workers in the same facility supports this hypothesis [111].

## 11. Conclusions

The SARS-CoV-2 pandemic is one of the greatest challenges in medicine and health care in recent decades. Various studies have demonstrated similarities between SARS-CoV-2 and SARS-CoV: both viruses utilize ACE2 and share the same pathway of host cell infection, resulting in a comparable disease pattern. Thus, findings on SARS-CoV are at least partially extrapolatable to SARS-CoV-2. Encouraging results of in vitro experiments with hydroxychloroquine have suggested that lysosomotropic compounds may be used as tools in fighting SARS-CoV-2 infections, by potentially triggering a variety of cellular modifications that impede alveolar cell infection and viral replication in this context.

Modulating the effects of lysosomotropic compounds on cytokines and interleukins is likely to provide a means of preventing the development of CRS/cytokine storm and, concomitantly, the rapid, severe, and serious deterioration in SARS-CoV-2 infection. Clinically approved lysosomotropic compounds are used in various medical applications and might be selected according to individual characteristics. With glycopeptide antibiotics such as teicoplanin, approved and available lysosomotropic compounds may prevent both viral and secondary bacterial infections. Therefore, for all lysosomotropic active compounds intended to be used off-label, attention must be paid to the pharmacological effects of their common indications and, if necessary, the administration route should be switched to a local one (e.g., inhalation). Timely application might aid in preventing viral infections with SARS-CoV-2 as well as severe progression.

Lysosomotropic compounds appear to display the more versatile and thus more promising prophylaxis and treatment approach for SARS-CoV-2 infection/COVID-19 as they are intended to target various events during infection and in the disease process. Such active compounds provide a valuable backbone upon which a personalized therapy can be tailored by combination with TMPRSS2 inhibitors, antibodies, antibiotics, and remdesivir.

Patients already receiving lysosomotropic compounds, e.g., sertraline, terbinafine, terfenadine, or trimipramine for pre-existing conditions at appropriate dosages might be protected against viral infections.

Evidence indicates that in patients with skin disorders, such as psoriasis or atopic dermatitis, the lysosomal pH is increased. Therefore, these individuals are presumed to be poor vectors, because conveying infection may be difficult.

The findings from various scientific fields jointly support a model from which a treatment strategy may be developed. The considerations described here await further support from statistical analysis of patient data. If further confirmed, the results may contribute another option for preventing and treating SARS-CoV-2 infections/COVID-19.

## Figures and Tables

**Table 1:** Variety of approved lysosomotropic compounds for various indications [36,41,42].

Drug Class	Lysosomotropic Drug	
Antidepressants (tricyclic)	Amitriptyline (++) Imipramine (++) Trimipramine (++) Maprotiline (++) **Clomipramine** ^# **x**^/***	Nortriptyline (++) Desipramine (++) Doxepine (++) Protriptyline (+)
Antidepressants (SSRI)	Fluoxetine (+) Setraline (++)	Norfluoxetine (+) Paroxetine (+)
Antimycotics	Terbinafine ^#^ (++)	
Antipsychotics	**Chlorpromazine^x/xx^** (++) Promazine (++)	Levomepromazine (++) **Promethazine^x^** (+)
Neuroleptics	Chlorprothixene (++) Thioridazine (++) **Thiothixene ^x^**/***	Perazine (++) Triflupromazine ^x/^** Fluspirilene ^x^/^##^
Tyrosine kinase inhibitors	**Imatinib **^#^**^x^**/***	**Dasatinib **^#^**^x^**/***
Calcium channel blockers	Amlodipine (-)	
Antirheumatics (antiprotozoals)	**Chloroquine^x/^^xx^** (++) **Mefloquine **^#o^**^x^**/***	**Hydroxychloroquine^x/^^xx^** (++)
Ovulation inducers	Clomiphene (++)	
Estrogen Receptor Antagonist	**Toremifene ^#^^x^**/***	
H_1_-antihistaminics	**Astemizole^x^** *	Terfenadine (++)
Anticholinergics (H_1_-antihistaminics)	**Benzatropine^x^** (-),	
Antibiotics (glycopeptides)	**Teicoplanin^x^**/*** **Oritavancin^x^**/***	**Dalbavancin^x^**/*** **Telavancin^x^**/***

Achievement of the desired lysosomotropic effect depends on the active compound, the dosage, and accumulation in lysosomes. Unless indicated, maximum daily doses are split into three applications. Lysosomal drug concentration (effect) within the therapeutic margin in vivo (expected): (++) occurs at maximum daily dosage and is very likely in low or initial dosage, (+) very likely at maximum daily dosage and is possible in low or initial dosage, (o) possible at maximum daily dosage and unlikely in low or initial dosage, and (-) unlikely even at maximum daily dosage; * withdrawn from the market (in most countries); ** veterinary use only; dosage: ^#^ single dose per day, ^#o^ dosage depending on treatment or prophylaxis (of malaria); **^x^in vitro anti SARS-CoV** tested, **^xx^in vitro anti SARS-CoV-2** tested [28,29,36,41,42,45,131,132]; *** lysosomotropism very likely, but not yet confirmed, lysosomal drug concentration (effect) within the therapeutic margin expected; ^##^ lysosomotropism very likely, but not yet confirmed, no lysosomal drug concentration (effect) within the therapeutic margin expected.

## Abbreviations

ACE2	Angiotensin-converting enzyme 2
ARDS	acute respiratory distress syndrome
BID	twice per day
COVID-19	Coronavirus disease 2019
CRS	cytokine release syndrome
DIC	disseminated intravascular coagulation
DMARD	disease-modifying antirheumatic drug
FA	fatty acid
G6PD	Glucose-6-phosphate dehydrogenase deficiency
HAT	human airway trypsin-like protease
HIV	human immunodeficiency viruses
IL-1R	IL-1 receptor
IL-6R	IL-6 receptor
ORF	open reading frame
RdRp	RNA dependent RNA polymerase
SSRI	selective serotonin reuptake inhibitors
V-ATPase	vacuolar H^+^-ATPase
